# Preoperative Prediction of Malignant Transformation of Sinonasal Inverted Papilloma Using MR Radiomics

**DOI:** 10.3389/fonc.2022.870544

**Published:** 2022-03-23

**Authors:** Yang Yan, Yujia Liu, Jianhua Tao, Zheng Li, Xiaoxia Qu, Jian Guo, Junfang Xian

**Affiliations:** ^1^ Department of Radiology, Beijing Tongren Hospital, Capital Medical University, Beijing, China; ^2^ School of Artificial Intelligence, University of Chinese Academy of Sciences, Beijing, China; ^3^ Chinese Academy of Sciences Key Laboratory of Molecular Imaging, Institute of Automation, Chinese Academy of Sciences, Beijing, China

**Keywords:** inverted papilloma (IP), squamous cell carcinoma, sinonasal cancer, radiomics, magnetic resonance imaging

## Abstract

**Purpose:**

Accurate preoperative prediction of the malignant transformation of sinonasal inverted papilloma (IP) is essential for guiding biopsy, planning appropriate surgery and prognosis of patients. We aimed to investigate the value of MRI-based radiomics in discriminating IP from IP-transformed squamous cell carcinomas (IP-SCC).

**Methods:**

A total of 236 patients with IP-SCC (n=92) or IP (n=144) were enrolled and divided into a training cohort and a testing cohort. Preoperative MR images including T1-weighted, T2-weighted, and contrast enhanced T1-weighted images were collected. Radiomic features were extracted from MR images and key features were merged into a radiomic model. A morphological features model was developed based on MR morphological features assessed by radiologists. A combined model combining radiomic features and morphological features was generated using multivariable logistic regression. For comparison, two head and neck radiologists were independently invited to distinguish IP-SCC from IP. The area under the receiver operating characteristics curve (AUC) was used to assess the performance of all models.

**Results:**

A total of 3948 radiomic features were extracted from three MR sequences. After feature selection, we saved 15 key features for modeling. The AUC, sensitivity, specificity, and accuracy on the testing cohort of the combined model based on radiomic and morphological features were respectively 0.962, 0.828, 0.94, and 0.899. The diagnostic ability of the combined model outperformed the morphological features model and also outperformed the two head and neck radiologists.

**Conclusions:**

A combined model based on MR radiomic and morphological features could serve as a potential tool to accurately predict IP-SCC, which might improve patient counseling and make more precise treatment planning.

## Introduction

Inverted papilloma (IP) is an uncommon sinonasal epithelial neoplasm accounting for 0.5% to 4.0% of all primary sinonasal neoplasms (1), and it is characterized by aggressive behavior, high recurrence rate, and a 7% to 10% possibility of malignant transformation into squamous cell carcinoma (IP-SCC) (2). The incidence of IP associated with the synchronously or metachronous SCC is reported from 2% to 53% (2–5), in which synchronous SCC accounts for approximately 55%–70% (2, 5, 6). Therefore, it is essential for precision diagnosis and treatment as well as prognosis to accurately predict IP-SCC preoperatively (3, 7, 8). However, it is very difficult to diagnose IP-SCC preoperatively due to similarity in clinical presentation and imaging findings with IP (9).

Local biopsy based on endoscopy is the most common surveillance technique (10). However, it is difficult for surgeons to inspect areas in the sinus that are not easily seen, and the accuracy of biopsies may be affected by sampling errors (11). Some studies have found that pain, epistaxis, and recurrence are the clinical presentations of malignant transformation of IP (12–14). However, these clinical presentations could also be found in IP patients, and sample sizes of IP-SCC in these studies are relatively small (5).

CT and MRI have also been used to distinguish IP-SCC from IP. Although IP-SCC on CT scan can show significantly higher bone destruction (13), this finding is quite nonspecific because IP may also have aggressive bone destruction (15, 16). So far, MRI has more promise in detecting malignant transformation. Convoluted cerebriform pattern (CCP) has been proved as a classical and reliable MRI feature of IP (17–19). Several studies have reported that focal loss of CCP may indicate malignant transformation of IP (18, 20, 21). Recently, some studies have attempted to predict IP-SCC using the loss of CCP combined with MR morphological features associated with malignancy, and achieved high specificity, but the sensitivity was unsatisfactory (9, 22). This is mainly because designation of CCP is relatively subjective and may be affected by radiologists’ misinterpretation, particularly when evaluating smaller tumors (9). Therefore, it is necessary to develop a more objective form of image analysis.

Radiomics is a novel technique which extracts large-scale quantitative features from medical images and constructs machine learning models based on these features (23–26). Radiomics has been widely used in cancer screening, diagnosis, treatment, and outcome prediction (27–30). Recently, Ramkumar et al. (31) found that MRI-based texture analysis had the potential to differentiate SCC from IP. However, there is no study investigating the application of radiomics to the differentiation of IP and IP-transformed squamous cell carcinomas.

Therefore, this study aimed to investigate the value of MRI-based radiomics in discriminating sinonasal IP from IP-transformed squamous cell carcinomas, and to improve the accuracy of preoperative diagnosis of IP-SCC.

## Materials and Methods

### Patients

**Figure 1:**
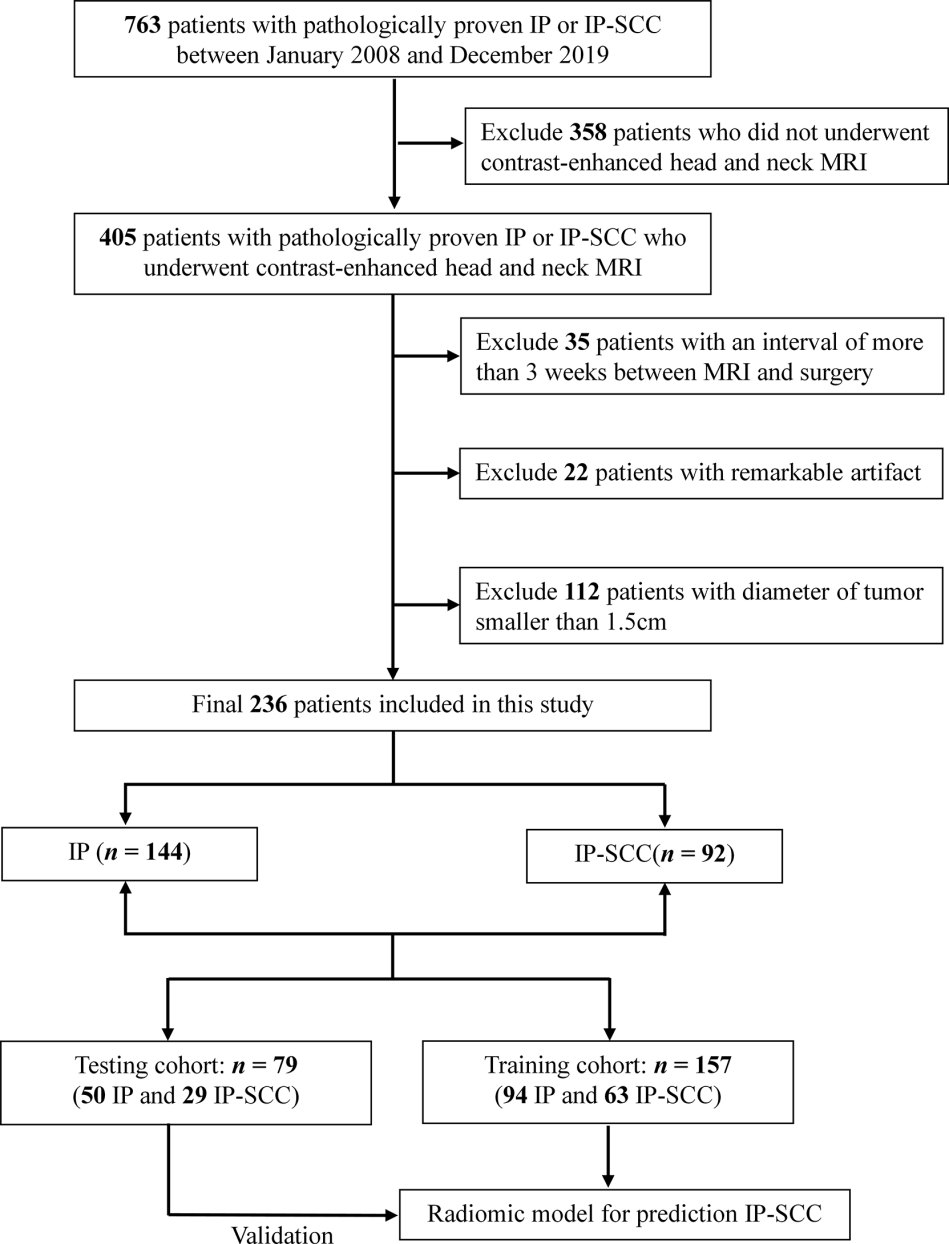
Flow diagram shows the procedure of data selection for prediction of malignant transformation of inverted papilloma. IP, inverted papilloma; IP-SCC, inverted papilloma–transformed squamous cell carcinoma.

This retrospective study has been approved by our institutional review board and the informed consents were waived. The medical records of pathologically proven IP or IP-SCC patients who underwent surgery at Beijing Tongren Hospital were retrospectively reviewed between January 2008 and December 2019. Recurrent tumors were included in the study. Patients were required to have a contrast-enhanced head and neck MRI within 3 weeks before surgical resection. Patients were excluded if they had chemotherapy or radiation therapy. In addition, patients were further excluded if the maximum diameter of the tumor was smaller than 1.5 cm on axial MR slices to ensure that enough radiomics features could be extracted. Finally, 236 patients with IP-SCC (n=92) or IP (n=144) were included in the study. The patients were randomly divided into a training cohort (n=157) and a testing cohort (n=79) at a ratio of 2:1 (**Figure 1**).

Demographic and clinical data were collected from enrolled patients, including gender, age, tumor location, and history of IP resection.

### Image Data Acquisition

All MR images were obtained with 3.0 T MR scanners (Ingenia; Philips Healthcare, Amsterdam, The Netherlands; or GE Signa HDxt, GE Healthcare, Milwaukee, USA; or GE Discovery 750, GE Healthcare, Milwaukee, USA) using an 8-channel head coil. The MR protocol included axial fast spin-echo (FSE) T1-weighted images (T1WI), FSE T2-weighted images (T2WI), and fat-saturated contrast enhanced T1WI (CE-T1WI). Contrast-enhanced T1-weighted images (CE-T1WI) were obtained after the intravenous bolus injection of 0.1 mL/kg gadopentetate dimeglumine. The detailed parameters for MR images acquisition are shown in **Table S1**.

### Images Segmentation

A head and neck radiologist (Y.Y.) segmented the Region of Interest (ROIs) of tumor for all of the patients using ITK-SNAP software (www.itksnap.org). For each MR sequence, the slice with the largest tumor area and its adjacent slices were selected and the outline of the tumor was delineated. In order to check the intra-reader agreement of tumor segmentation, we randomly selected 30 patients and segmented the ROI again by another radiologist (JH.T.). The intra-class correlation coefficient (ICC) was calculated to measure the robustness of the radiomic features.

### Feature Selection and Radiomic Model Construction

Before feature selection, all MR images were normalized and resampled using B-spline interpolation to 1mm×1mm×1mm in order to compensate for scanner-dependent variability in image intensity. Detailed process shown in **Supplementary A1**. A total of 1316 radiomic features were extracted from each MR sequence using Pyradiomics (https://pyradiomics.readthedocs.io). For each ROI, the shape, intensity (first order statistics), and textural features were calculated and normalized by the z-score method (29, 32). Then, we respectively constructed three single-sequence radiomic signatures (T1WI, T2WI, and CE-T1WI) using corresponding sequence images.

The building process of each single-sequence signature was as follows: (1) we retained features that were significantly associated with IP-SCC in a univariable analysis; (2) the features with good robustness (ICC>0.75) were reserved for further analysis (33); (3) the minimum redundancy maximum relevance (mRMR) method was used to rank the radiomic features with mutual information. The top five features were selected as the final radiomic features; (4) the radiomic signatures were generated using logistic regression based on the five features.

We further combined the three single-sequence radiomic signatures to construct a radiomic model using multivariable logistic regression.

### Morphological Model and Combined Model Construction

The morphological features assessment was performed independently by two head and neck radiologists (Y.Y. 5 years of clinical experience and JH.T. 10 years of clinical experience, respectively) who were blinded to histopathology and clinical information. Morphological features included cranial base invasion, orbit invasion, soft tissue invasion in the maxillofacial area, internal necrosis of the tumor, and loss of a convoluted cerebriform pattern (CCP). We designated a CCP as alternating hyperintense and hypointense bands in the solid components of the tumor on T2WI or CE-T1WI. The loss of CCP was categorized as partial, total, and no loss of CCP (9, 34). Cohen’s kappa interrater reliability score was calculated to measure the inter-group agreement of the two radiologists’ assessments for the morphological features.

For the morphological model, we first selected the morphological features that were of significant relevance to IP-SCC. Then, based on significant features, we respectively used two classifiers, including logistic regression and support vector machine (SVM), to construct and save the one with better diagnosis ability as a morphological model.

Finally, we built a combined model that embedded the radiomic model and morphological model. Linear regression was used for the merging of the two models.

### Model Validation and Comparison

The performances of the prediction models were assessed using the receiver operating characteristic (ROC) curve and area under the curve (AUC) with a 95% confidence interval (CI). The sensitivity, specificity, and accuracy were also calculated for further evaluation. The calibration curves were applied to modify and reduce the bias of the models. Furthermore, the performance of the constructed models in this study were evaluated by a 5-fold cross-validation setup.

For comparison, a senior and junior head and neck radiologist (Y.Y. and JH.T.) were invited to independently diagnose IP-SCC *via* MR images. Both of the radiologists were blinded to the histopathology during the diagnosis process.

All of the images were scanned by three MR scanners (Philips Ingenia; GE Signa HDxt; GE Discovery 750). Stratified analysis was used to check whether the models were affected by different MR scanners.

### Statistical Analysis

The chi-square test and two-tailed *t* tests were used to calculate univariate analyses for categorical and continuous variables, respectively. *P*<0.05 was considered statistically significance. Radiomic feature standardization, selection and model building were performed using the Python (version: 3.7). The radiomic features with ICC > 0.75 were regarded as having good robustness and stability. ROC analysis was used to evaluate the diagnostic performances of models and radiologist assessment [95% confidence intervals (CIs), specificity, and sensitivity were also calculated]. The DeLong test was performed to compare the diagnostic performance of models. The statistical analyses were performed with R software (version: 3.6) and Python (version: 3.7).

## Results

### Clinical and Morphological Characteristics

There were 157 patients in the training cohort (94 IPs and 63 IP-SCCs). The remaining 79 patients were allocated into the testing cohort (50 IPs and 29 IP-SCCs). As shown in **Table 1**, the average ages of patients for IP-SCC and IP were 57.4 and 51.6 years, respectively (*P* = 0.001). There was no difference in sex and prior IP resection between IP-SCC and IP. The maxillary sinus was a more common tumor site in IPs than IP-SCCs (*P* <0.001); the frontal sinus was a more common tumor site in IP-SCCs than IPs (*P* < 0.001).

**Table 1:** Demographics and Clinical Characteristics.

	IP-SCC (n = 92)	IP (n = 144)	P
Age, years (mean ± SD)	57.4 ± 13.6	51.6 ± 11.8	0.001
Sex, *n* (%)			0.070
Male	72 (78.3)	97 (67.4)	
Female	20 (21.7)	47 (32.6)	
Tumor location*, *n* (%)			
Nasal cavity	54 (58.7)	100 (69.4)	0.091
Maxillary sinus	55 (59.8)	126 (87.5)	<0.001
Ethmoid sinus	40 (43.5)	55 (38.2)	0.419
Sphenoid sinus	4 (4.3)	3 (2.1)	0.322
Frontal sinus	22 (23.9)	10 (6.9)	<0.001
Prior IP resection, *n* (%)			0.604
Yes	39 (42.4)	66 (45.8)	
No	53 (57.6)	78 (54.2)	

^*^Multiple locations of tumors were counted separately.

IP, inverted papilloma; IP-SCC, inverted papilloma–transformed squamous cell carcinoma.

The Cohen’s kappa interrater reliability score calculated for five MR morphological features assessed by two radiologists were over 0.8, reflecting a strong agreement (**Table S2**) **(** 35). As shown in **Table 2**, there were five features that were significantly associated with IP-SCC in the training cohort including cranial base invasion (*P* = 0.001), orbit invasion (*P* <0.001), soft tissue invasion in the maxillofacial area (*P* = 0.003), internal necrosis of the tumor (*P* = 0.003), and loss of a convoluted cerebriform pattern (*P* < 0.001). All of these features were also significant in the testing cohort.

**Table 2:** Morphological features of patients in training and Testing cohorts.

	Training cohort (n=157)			Testing cohort (n=79)		
	IP-SCC (n = 63)	IP (n = 94)	P	IP-SCC (n = 29)	IP (n = 50)	P
Internal necrosis of the tumor, *n* (%)			0.002			0.007
Absent	39 (61.9)	79 (84)		19(65.5)	45 (90)	
Present	24 (38.1)	15 (16)		10(34.5)	5 (10)	
Orbit invasion, *n* (%)			<0.001			<0.001
Absent	40 (63.5)	90 (95.7)		19(65.5)	50 (100)	
Present	23 (36.5)	4 (4.3)		10(34.5)	0 (0)	
Cranial base invasion, *n* (%)			0.001			0.020
Absent	53 (84.1)	93 (98.9)		26 (89.7)	50 (100)	
Present	10 (15.9)	1 (1.1)		3(10.3)	0(0)	
Soft tissue invasion in the maxillofacial area, *n* (%)			0.001			<0.001
Absent	49 (77.8)	89 (94.7)		21 (72.4)	50(100)	
Present	14 (22.2)	5 (5.3)		8 (27.6)	0 (0)	
Loss of CCP, *n* (%)			<0.001			<0.001
Absent	26 (41.3)	83 (88.3)		9 (31)	45 (90)	
Partial	27 (42.8)	10 (10.6)		8 (27.6)	5 (10)	
Total	10 (15.9)	1 (1.1)		12 (41.4)	0 (0)	

IP, inverted papilloma; IP-SCC, inverted papilloma–transformed squamous cell carcinoma; CCP, convoluted cerebriform pattern.

### Reproducibility and Feature Selection

A total of 3948 features (1316 features per MR sequence) were extracted from the tumor ROIs. After the univariable analysis, we selected 701, 757, and 748 features with a significant difference from T1WI, T2WI, and CET1WI, respectively. There were 580 (82.8%), 714 (94.4%), and 645 (86.2%) features showing good consistency and robustness from T1WI, T2WI and CE-T1WI sequences, respectively. For each sequence, we finally selected five key features to construct a radiomic signature. **Table S3** shows those key features in the three sequences.

### Diagnostic Performances of Radiomic and Morphological Models

The performances of the three single-sequence signatures are shown in **Table S4**. In the validation cohort, the AUC of the CE-T1WI-signature (AUC = 0.931) surpassed the T1WI-signature (AUC = 0.857) and T2WI-signature (AUC = 0.886).

A radiomic model was constructed by fusing a T1WI-signature, T2WI-signature, and CE-T1WI-signature. As shown in **Table 3**, the radiomic model had good ability in discriminating IP-SCC from IP. In the testing cohort, the AUC of the radiomic model (AUC = 0.940) was better than the three single-sequence signatures.

**Table 3:** The Performance of Models in Training and Testing cohorts.

Model	AUC (95%CI)	SEN	SPE	ACC	TP	FN	FP	TN
Morphological features model								
Training cohort	—	0.667	0.883	0.796	42	21	11	83
Testing cohort	—	0.690	0.9	0.823	20	9	5	45
Radiomic model								
Training cohort	0.954 (0.926-0.982)	0.857	0.883	0.873	54	9	11	83
Testing cohort	0.940 (0.888-0.992)	0.793	0.92	0.873	23	6	4	46
Combined model								
Training cohort	0.957 (0.928-0.987)	0.889	0.915	0.904	56	7	8	86
Testing cohort	0.962 (0.927-0.997)	0.828	0.94	0.899	24	5	3	47
Senior radiologist								
Training cohort	—	0.571	0.947	0.796	36	27	5	89
Testing cohort	—	0.517	0.980	0.810	15	14	1	49
Junior radiologist								
Training cohort	—	0.492	0.904	0.739	31	32	9	85
Testing cohort	—	0.448	0.960	0.772	13	16	2	48

AUC, area under the curve; CI, confidence interval; SEN, Sensitivity; SPE, Specificity; ACC, Accuracy; TP, True Positive; FN, False Negative; FP, False Positive; TN, True Negative.

We chose SVM classifier to construct morphological features model containing five morphological features. The morphological features model reached accuracy of 0.796 and 0.823 in training and testing cohorts, respectively (**Table 3**).

### Diagnostic Performances of the Combined Model and Comparison With Radiologists

**Figure 2:**
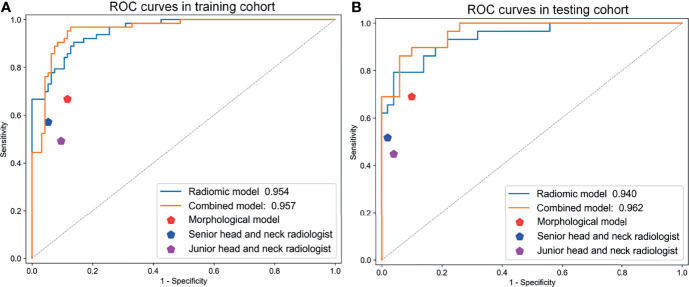
The ROC curves of the radiomic model, morphological features model, and combined model in **(A)** training and **(B)** testing cohorts.

The ROC curves of the radiomic model, and combined model are shown in **Figure 2**. The quantitative indices of the models are shown in **Table 3**. The AUC, sensitivity, specificity, and accuracy on the test cohort of the combined model were respectively 0.962, 0.828, 0.94, and 0.899. The sensitivity, specificity, and accuracy of the combined model surpassed morphological features model.

**Figure 3:**
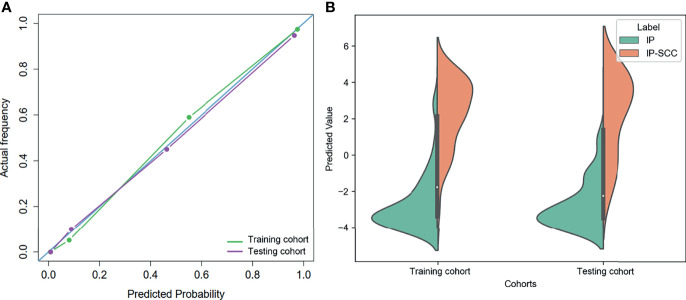
**(A)** The calibration curves of the combined model in training and testing cohorts. **(B)** Distribution of the combined model predicted values in training and testing cohorts.

We also randomly divided the dataset into five parts. After 5-fold cross-validation, we noticed that the AUCs of combined model range from 0.964-0.9790 in training cohort and 0.932-0.985 in testing cohort. The average AUC of combined model (training cohort:0.971; testing cohort: 0.951) surpassed than that of other models **(** **Table S5** **)**. As shown in **Figure 3**, the calibration curves demonstrated that the predicted results of the combined model were in good agreement with the actual results.

**Figure 4:**
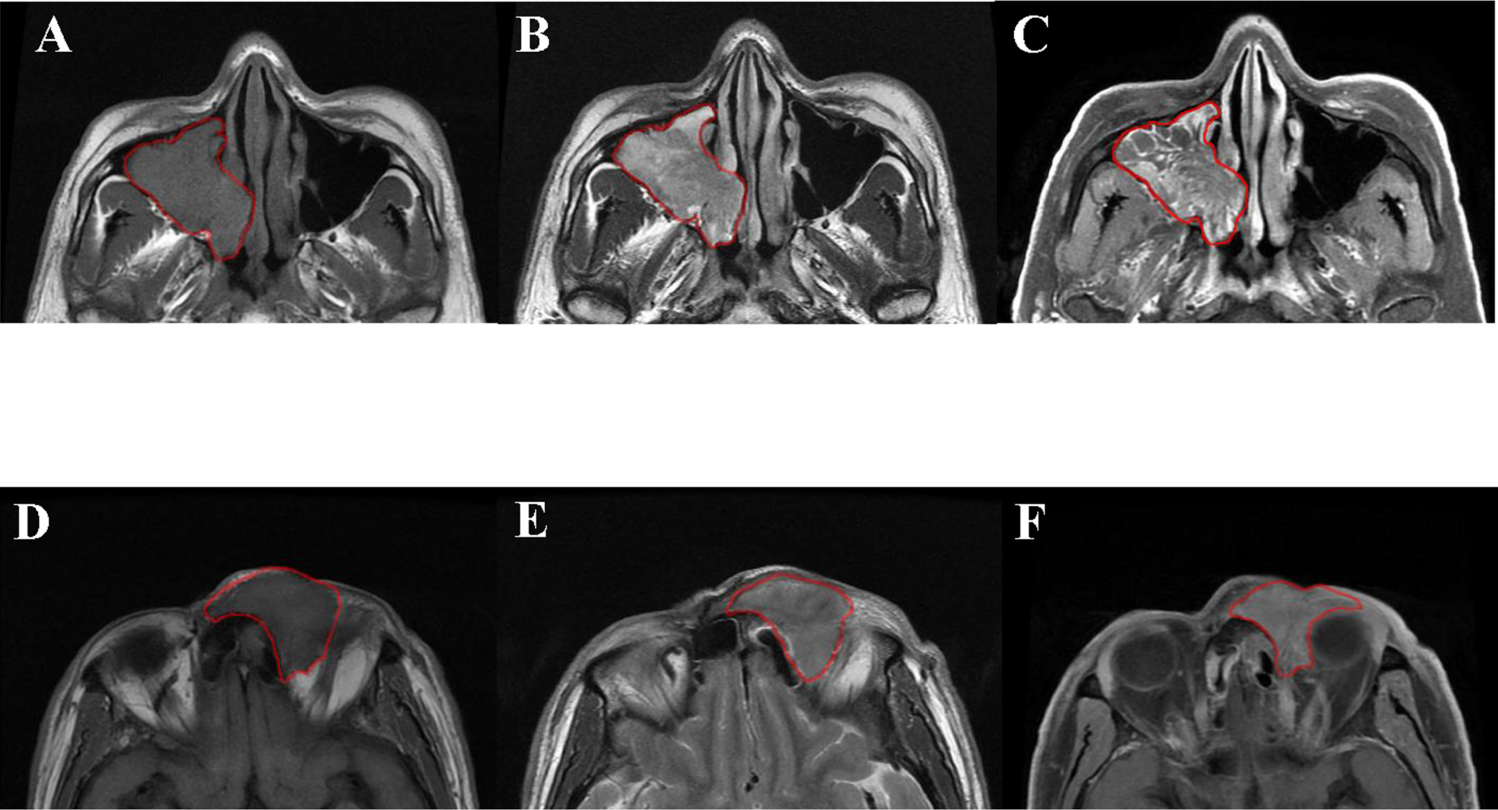
Patient 1: axial T1WI **(A)**, axial T2WI **(B)**, and axial contrast-enhanced T1WI **(C)**. A 48-year-old man was pathologically diagnosed as inverted papilloma in the right nasal cavity and maxillary sinus, with severe epithelial atypical hyperplasia and carcinogenesis. The presence of convoluted cerebriform pattern and absence of extra-sinonasal involvement led to a misclassification as benign by the two radiologists, whereas the radiomic model well-classified it as malignant. Patient 2: axial T1WI **(D)**, axial T2WI **(E)**, and axial contrast-enhanced T1WI **(F)**. A68 year-old man was pathologically diagnosed as an inverted papilloma in the left frontal sinus and, with squamous cell carcinoma in some areas. In this case, orbital invasion was a key feature of malignancy easily seen by radiologists. The two radiologists well-classified the case as malignant, whereas the radiomic model misclassified it as benign.

We also compared the performance of the two head and neck radiologists and the combined model (**Figure 4**). As shown in **Table 3**, the combined model had better accuracy (0.899) and sensitivity (0.828) than the two radiologists in the testing cohort. The specificity of the combined model was similar to the two radiologists. This indicated that the combined model could diagnose more patients with IP-SCC, while avoiding missed diagnoses.

### Impact of Different MR Scanners on the Models

**Figure 5:**
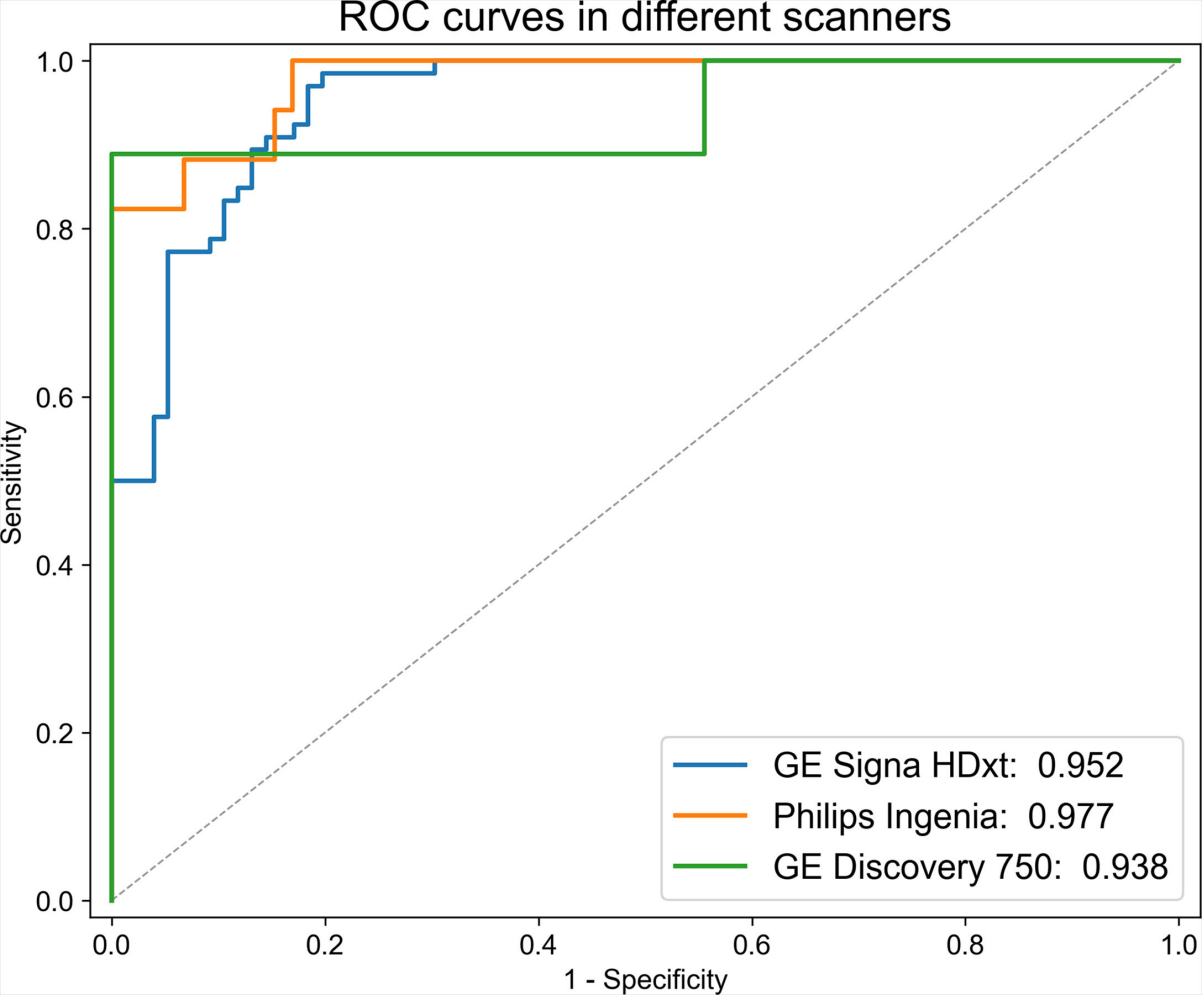
ROC curves of the combined model in different MR scanners. There was no significant difference in the performance of the combined model among the three MR scanners.

In the stratified analysis, all patients were divided into three subgroups according to different MR scanners (Philips Ingenia; GE Signa HDxt; GE Discovery 750). From **Table S6**, we found that the radiomics features in the model performed well in different scanners. As shown in **Figure 5**, there was no significant difference in the performance of the combined model among the three MR scanners (*P* ≥ 0.249).

## Discussion

Accurate prediction of the malignant transformation of IP has long been a focus of clinical concern and challenge (1, 5). As malignant tumors require more extensive surgical resection and adjuvant treatment, preoperatively prediction of the malignant transformation of IP is critical for improving patient counseling and planning precise treatment (3).

In this retrospective study, we developed a combined model based on MR radiomic features and morphological features to discriminate IP-SCC from IP. The results showed that the combined model had excellent diagnostic accuracy, which performed better than the radiomic model and morphological model. Moreover, the combined model outperformed the two head and neck radiologists, demonstrating the potential clinical value of the model.

In previous studies, many researchers found that the loss of a convoluted cerebriform pattern (CCP) was a significant MRI feature for predicting IP-SCC (18, 20–22, 34). Yan et al. (9) previously evaluated 35 IP-SCC patients and found that 60% of patients had a complete loss of CCP. In the study of Zhang et al. (22), the sensitivity and specificity of loss of CCP for predicting IP-SCC were 73.4% and 85.4%, respectively. In our study, the loss of CCP was found in 61.9% (57/92) of IP-SCC patients and 11.1% (16/144) of IP patients. These studies suggested that it is not sufficient for differentiating IP-SCC from IP only by the presence or absence of CCP. The potential possibility of interpretive error increases, particularly when a small focus of SCC exists within a large benign IP and cannot be recognized because of the presence of CCP (22, 36).

In terms of evaluating the intrinsic appearance of a tumor, radiomics has a strong ability of feature extraction and texture analysis. It can provide incremental benefits when the radiologists’ visual recognition ability reaches its limit, and can comprehensively extract and analyze the internal fine structure of the tumor (37–39). In the current study, the best 15 radiomic features that could discriminate IP-SCC from IP included 13 texture features, 1 shape feature, and 1 intensity feature. Among them, the texture features which reflect gray-level nonuniformity had higher values in IP-SCC, which may be explained by a higher heterogeneity of the images. Accurately assessing the heterogeneity of a small region would be the key to detecting focal malignant transformation in IP (31).

Interestingly, radiomics could also extract useful features from images that may be ignored by the human eyes. The study of Ramkumar et al. (31) found that the texture features extracted from non-enhanced T1WI could be used to distinguish IP from SCC. To our best knowledge, the value of T1WI in distinguishing malignant transformation of IP has not receive attention. Therefore, our study selected radiomic features from T1WI, T2WI, and CE-T1WI images to avoid missing key features. The results showed that the key radiomic features of these three sequences had good diagnostic performances. The features in the CE-T1WI sequence showed the best diagnostic performance. In this study, we fused the radiomic features from the three sequences together to construct a radiomic model, which has a powerful ability to identify IP-SCC.

However, the radiomic model we constructed was meant to supplement radiologists’ diagnostic abilities rather than compete with them. In clinics, the diagnosis is based on the synthesis of all available data, including not only the intrinsic appearance of tumor, but also the imaging features of the peritumoral environment, such as the invasion and destruction of the tumor in the surrounding tissues (31). Therefore, five MR morphological features assessed by radiologists were analyzed in this study, these features were significantly different between IP and IP-SCC. Among them, cranial base invasion, orbit invasion, and soft tissue invasion in the maxillofacial area reflect the involvement of peritumoral tissues, whereas these features were not common in IP-SCCs of our study. Some malignancies of this study, including 29 cases of pathologically confirmed carcinoma *in situ*, did not show obvious aggressiveness, leading to the high specificity but unsatisfactory sensitivity of diagnostic results by the two head and neck radiologists who give results based on these morphological features.

The model based on morphological features performed worse than the radiomic model, the combined model (the combination of radiomic model and morphological feature model) performed better than the other two models. This indicated that the combined model might improve the limit of ignoring the peritumoral environment produced by radiomic model, and would bring an icing on the cake effect.

Our study had some limitations. First, we did not include diffusion weighted images. It has been reported that the apparent diffusion coefficient (ADC) value can also distinguish IP-SCC from IP (9). However, if malignant transformation occurs as a small focus, the mean ADC value cannot represent IP-SCC in the background of IP (34), which may require a histogram analysis of ADC values for the whole tumor (34). Second, we included inverted papilloma-transformed squamous cell carcinoma as much as possible to develop a robust model, the current study included 105 recurrent patients, which may decrease the quality of data. But we have not found any difference in radiomic features between the recurrent tumors and primary tumors. The outcomes of a previous treatment (scar, hyperostosis, iatrogenic anatomical changes) could influence the radiological interpretation. However, scar and hyperostosis identified by MR images may not be totally consistent with histopathological results, so they are not evaluated in the current study, which my influence the performance of the model. We will investigate them in the future. Third, although the radiomic model can distinguish IP-SCC from IP, it cannot accurately pinpoint which regions develop malignant transformation within the tumor, further prospective studies were required to ensure that the histopathological analysis results can be accurately synchronized and corresponded with MR images (31). The goal of the next step for radiomics should be to assist diagnosis by highlighting the most suspicious regions of malignant transformation. Forth, the rate of SCC in inverted papillomas (39%) is higher than the trends reported in the literature, which may bring bias to the assessment of the diagnostic performance of the model. Our hospital is one of the best hospitals in otorhinolaryngology in China, many patients with IP-SCC seek treatment at our hospital, so the rate of SCC in inverted papillomas in higher than trends reported in the literature. We will validate and optimize the model using multicenter data in the future.

In conclusion, we constructed a combined model based on MR radiomic features and morphological features to discriminate IP-SCC from IP. The model could serve as a potential tool to assist clinicians for an accurate and noninvasive diagnosis of the malignant transformation in IP patients, which might improve patient counseling and help to make more precise treatment planning for IP-SCC.

## Data Availability Statement

The raw data supporting the conclusions of this article will be made available by the authors, without undue reservation.

## Ethics Statement

The studies involving human participants were reviewed and approved by The Ethics Review Committee of Beijing Tongren Hospital. Written informed consent for participation was not required for this study in accordance with the national legislation and the institutional requirements.

## Author Contributions

YY, YL, and JX designed and supervised the study. YY, ZL, and JG collected the clinical data, MR images, and histopathological results. YY, YL, and JT processed the clinical and images data. YY, YL, and XQ performed the statistical analysis. YY and YL drafted and revised the manuscript. All authors contributed to the article and approved the submitted version.

## Funding

This work was supported by the National Key R&D Program of China (2017YFA0205200, 2017YFC1308700, 2017YFA0700401, 2017YFC1309100), National Natural Science Foundation of China (82022036, 91959130, 81971776, 81771924, 62027901, 81930053, 81227901), Beijing Natural Science Foundation (L182061), Strategic Priority Research Program of the Chinese Academy of Sciences (XDB 38040200), Chinese Academy of Sciences under Grant (GJJSTD20170004 and QYZDJ-SSW-JSC005), Project of High-Level Talents Team Introduction in Zhuhai City (Zhuhai HLHPTP201703), Youth Innovation Promotion Association CAS (2017175), Beijing Municipal Administration of Hospitals’Ascent Plan (DFL20190203), Beijing Municipal Administration of Hospitals Clinical Medicine Development of Special Funding Support (ZYLX201704), and High Level Health Technical Personnel of Bureau of Health in Beijing (2014-2-005).

## Conflict of Interest

The authors declare that the research was conducted in the absence of any commercial or financial relationships that could be construed as a potential conflict of interest.

## Publisher’s Note

All claims expressed in this article are solely those of the authors and do not necessarily represent those of their affiliated organizations, or those of the publisher, the editors and the reviewers. Any product that may be evaluated in this article, or claim that may be made by its manufacturer, is not guaranteed or endorsed by the publisher.

## Abbreviations

IP, Inverted papilloma; IP-SCC, Inverted papilloma-transformed squamous cell carcinomas; CCP, Convoluted cerebriform pattern; T1WI, T1-weighted images; T2WI, T2-weighted images; CE-T1WI, Contrast-enhanced T1-weighted images; ROI, Regions of interest; ICC, intra-class correlation coefficient; mRMR, Minimum redundancy maximum relevance; SVM, Support vector machine; ROC, The area under the receiver operating characteristic; AUC, The area under the receiver operating characteristic curves; CI, confidence interval.
