# Supplementary material for: Preoperative Prediction of Malignant Transformation of Sinonasal Inverted Papilloma Using MR Radiomics
Source: Front Oncol. 2022 Mar 23;12:870544. doi: 10.3389/fonc.2022.870544 (PMC8983836; doi:10.3389/fonc.2022.870544)
Supplement: Supplementary file 1 [file DataSheet_1.docx]

**Preoperative** **prediction of malignant transformation of sinonasal inverted papilloma using MR** **radiomics**

**Supplementary A1: Feature extraction**

To reduce the effect of slice thickness variation, all images were resampled to the voxel size of 1mm×1mm×1mm 3 using B-Spline interpolation. To minimize the MRI intensity variations, we normalized the intensity of the image using the following formula (where $x$ indicates the original intensity; $f(x)$ indicates the normalized intensity; $\mu_{x}$ refers to the mean value of the image intensity values; $\sigma_{x}$ indicates the standard deviation of the image intensity values; $s$ is an optional scaling, by default, it is set to 1). Normalizes the image by centering it at the mean with standard deviation. Normalization is based on all gray values in the image, not just those inside the segmentation.

$$f(x)=\frac{s(x-\mu_{x})}{\sigma_{x}}$$

**Supplementary TABLE S1. |** MR scanning parameters.

| **MR Scanner** | **Sequence** | **TR**  **(ms)** | **TE**  **(ms)** | **FOV**  **(cm^2^)** | **Number**  **of slices** | **Slice**  **thickness**  **(mm)** | **Slice gap**  **(mm)** | **NEX** | **Matrix** |
| --- | --- | --- | --- | --- | --- | --- | --- | --- | --- |
| GE 3.0T  (Discovery 750) | T1WI FSE | 560–590 | 10 | 22 × 22 | 24 | 4-4.5 | 0.3-0.4 | 2 | 288 × 224 |
|  | T2WI FSE | 3975–4720 | 85-90 | 22 × 22 | 24 | 4-4.5 | 0.3-0.4 | 2 | 320 × 224 |
|  | CE-T1WI | 560–590 | 7-8 | 22 × 22 | 24 | 4-4.5 | 0.3-0.4 | 2 | 288 × 224 |
| GE 3.0T  (Signa HDxt) | T1WI FSE | 400–500 | 10 | 22 × 22 | 20 | 4-5 | 0.5 | 2 | 320 × 256 |
|  | T2WI FSE | 3500–4000 | 90 | 22 × 22 | 20 | 4-5 | 0.5 | 2 | 512 × 256 |
|  | CE-T1WI | 400-500 | 10 | 22× 22 | 20 | 4-5 | 0.5 | 2 | 320 × 224 |
| Philips 3.0T  (Ingenia) | T1WI TSE | 600–700 | 6-7 | 21 × 19 | 20 | 4 | 0.4 | 2 | 352 × 228 |
|  | T2WI TSE | 2500-3000 | 80-90 | 21 × 19 | 20 | 4 | 0.4 | 2 | 352 × 249 |
|  | CE-T1WI | 600–700 | 7-8 | 21 × 19 | 20 | 4 | 0.4 | 2 | 264 × 201 |

TR, repetition time; TE, echo time; FOV, Field of view; NEX, number of excitations; FSE, fast spin echo; TSE, turbo spin echo.

**Supplementary TABLE S2. |** The consistency test of morphological features assessed by the two radiologists.

| **Morphological features** | **kappa** |
| --- | --- |
| Internal necrosis of the tumor | 0.878 |
| Orbit invasion | 0.881 |
| Cranial base invasion | 0.820 |
| Soft tissue invasion in the maxillofacial area | 0.837 |
| Loss of CCP | 0.825 |

CCP, Convoluted Cerebriform Pattern.

**Supplementary TABLE S3. |** The key radiomics features in the radiomic model.

| **Radiomic signature** | **Radiomic feature** | **Types** |
| --- | --- | --- |
| T1WI-signature | squareroot_glszm_SizeZoneNonUniformity | Texture feature |
|  | wavelet.HLL_glrlm_RunEntropy | Texture feature |
|  | square_ngtdm_Coarseness | Texture feature |
|  | gradient_glszm_SizeZoneNonUniformityNormalized | Texture feature |
|  | original_shape_SurfaceVolumeRatio | Shape feature |
| T2WI-signature | logarithm_glcm_ClusterProminenc | Texture feature |
|  | logarithm_firstorder_Minimum | Intensity feature |
|  | logarithm_glszm_SizeZoneNonUniformity | Texture feature |
|  | logarithm_glcm_Contrast" | Texture feature |
|  | squareroot_ngtdm_Complexity | Shape feature |
| CE-T1WI-signature | logarithm_glszm_SizeZoneNonUniformity | Texture feature |
|  | logarithm_glcm_DifferenceVariance | Texture feature |
|  | square_firstorder_Minimum | Intensity feature |
|  | logarithm_glszm_LargeAreaLowGrayLevelEmphasis | Texture feature |
|  | logarithm_glszm_GrayLevelNonUniformityNormalized | Texture feature |

GLSZM, Gray-Level-Size-Zone-Matrix; GLRLM, Gray-Level-Run-Length-Matrix; NGTDM, Neighborhood gray-tone difference matrix; GLCM, Grey-Level-Co-occurrence-Matrix.

**Supplementary TABLE S4. |** The performance of the three single-sequence radiomic signatures in training and validation cohorts.

|  | **AUC (95%CI)** | **SEN** | **SPE** | **ACC** | **TP** | **FN** | **FP** | **TN** |
| --- | --- | --- | --- | --- | --- | --- | --- | --- |
| T1WI-signature |  |  |  |  |  |  |  |  |
| Training cohort | 0.906 (0.862-0.950) | 0.889 | 0.787 | 0.828 | 56 | 7 | 20 | 74 |
| Validation cohort | 0.857 (0.759-0.954) | 0.724 | 0.780 | 0.759 | 21 | 8 | 11 | 39 |
| T2WI-signature |  |  |  |  |  |  |  |  |
| Training cohort | 0.924 (0.885-0.963) | 0.794 | 0.894 | 0.854 | 50 | 13 | 10 | 84 |
| Validation cohort | 0.886 (0.805-0.966) | 0.759 | 0.92 | 0.861 | 22 | 7 | 4 | 46 |
| CE-T1WI-signature |  |  |  |  |  |  |  |  |
| Training cohort | 0.919 (0.879-0.960) | 0.778 | 0.872 | 0.834 | 49 | 14 | 12 | 82 |
| Validation cohort | 0.931 (0.875-0.987) | 0.759 | 0.94 | 0.873 | 22 | 7 | 3 | 47 |

AUC, area under the curve; CI, confidence interval; SEN, Sensitivity; SPE, Specificity; ACC, Accuracy; TP, True Positive; FN, False Negative; FP, False Positive; TN, True Negative.

**Supplementary TABLE S5. |** The AUCs of models by 5-fold cross-validation in training and testing cohorts.

|  | **1** | **2** | **3** | **4** | **5** | **Average** |
| --- | --- | --- | --- | --- | --- | --- |
| Radiomic model |  |  |  |  |  |  |
| Training cohort | 0.958 | 0.968 | 0.958 | 0.963 | 0.953 | 0.960 |
| Testing cohort | 0.934 | 0.899 | 0.925 | 0.904 | 0.976 | 0.928 |
| Combined model |  |  |  |  |  |  |
| Training cohort | 0.968 | 0.979 | 0.967 | 0.977 | 0.964 | 0.971 |
| Testing cohort | 0.932 | 0.940 | 0.962 | 0.936 | 0.985 | 0.951 |

**Supplementary TABLE S6. |** The key radiomics features values in different MR scanners.

| **Radiomic features** | **Philips Ingenia**  **(n=142)** | | | **GE Signa HDxt**  **(n=77)** | | | **GE Discovery 750**  **(n=18)** | | |
| --- | --- | --- | --- | --- | --- | --- | --- | --- | --- |
|  | **IP** | **IP-SCC** | **P** | **IP** | **IP-SCC** | **P** | **IP** | **IP-SCC** | **P** |
| **T1WI-signature** |  |  |  |  |  |  |  |  |  |
| squareroot_glszm_SizeZoneNonUniformity | -0.66±0.27 | 0.14±0.88 | ＜0.01 | -0.14±0.60 | 0.92±1.31 | ＜0.01 | -0.92±0.15 | -0.19±0.4 | ＜0.01 |
| wavelet.HLL_glrlm_RunEntropy | -0.95±0.74 | 0.04±0.56 | ＜0.01 | 0.03±0.60 | 0.71±0.8 | ＜0.01 | 0.05±0.62 | 1.12±0.67 | ＜0.01 |
| square_ngtdm_Coarseness | 0.16±1.63 | -0.02±0.26 | ＜0.01 | -0.08±0.02 | -0.09±0.01 | 0.02 | -0.06±0.04 | -0.08±0.01 | 0.04 |
| gradient_glszm_SizeZoneNonUniformityNormalized | -0.93±0.80 | 0.16±0.96 | ＜0.01 | -0.05±0.66 | 0.73±0.73 | ＜0.01 | 0.00±0.87 | 1.1±0.64 | 0.01 |
| original_shape_SurfaceVolumeRatio | 0.47±0.92 | -0.74±0.68 | ＜0.01 | 0.27±0.93 | -0.47±0.84 | ＜0.01 | 0.39±0.96 | -0.55±0.47 | 0.03 |
| **T2WI-signature** |  |  |  |  |  |  |  |  |  |
| logarithm_glcm_ClusterProminenc | -0.44±0.03 | -0.27±0.28 | ＜0.01 | -0.24±0.67 | 0.6±1.28 | ＜0.01 | -0.30±0.26 | 0.37±0.84 | 0.02 |
| logarithm_firstorder_Minimum | 1.17±0.89 | -0.48±0.52 | ＜0.01 | -0.52±0.20 | -0.68±0.19 | ＜0.01 | -0.06±0.99 | -0.63±0.33 | 0.19 |
| logarithm_glszm_SizeZoneNonUniformity | -0.71±0.13 | 0.14±0.96 | ＜0.01 | -0.31±0.58 | 0.88±1.21 | ＜0.01 | -0.60±0.13 | 0.30±0.64 | ＜0.01 |
| logarithm_glcm_Contrast" | -0.39±0.05 | -0.21±0.32 | ＜0.01 | 0.36±1.29 | 0.44±0.85 | ＜0.01 | -0.24±0.22 | 0.28±0.55 | 0.02 |
| squareroot_ngtdm_Complexity | -0.70±0.33 | 0.04±0.74 | ＜0.01 | 0.01±0.64 | 0.75±0.77 | ＜0.01 | -0.51±0.39 | 0.65±1.03 | 0.01 |
| **CE-T1WI-signature** |  |  |  |  |  |  |  |  |  |
| logarithm_glszm_SizeZoneNonUniformity | -0.52±0.20 | 0.19±0.56 | ＜0.01 | -0.42±0.28 | 0.81±1.41 | ＜0.01 | -0.51±0.22 | 0.62±1.00 | ＜0.01 |
| logarithm_glcm_DifferenceVariance | -0.31±0.58 | 0.06±0.7 | ＜0.01 | -0.39±0.15 | 0.63±1.54 | ＜0.01 | -0.34±0.32 | 1.62±2.13 | ＜0.01 |
| square_firstorder_Minimum | 0.36±1.29 | -0.39±0.15 | ＜0.01 | -0.01±0.58 | -0.45±0.07 | ＜0.01 | 0.81±1.91 | -0.46±0.02 | ＜0.01 |
| logarithm_glszm_LargeAreaLowGrayLevelEmphasis | 0.01±0.64 | -0.3±0.06 | ＜0.01 | 0.35±1.74 | -0.31±0.09 | ＜0.01 | 0.02±0.54 | -0.33±0.01 | ＜0.01 |
| logarithm_glszm_GrayLevelNonUniformityNormalized | 0.05±0.75 | -0.88±0.42 | ＜0.01 | 0.81±0.85 | -0.61±0.72 | ＜0.01 | 0.82±0.79 | -0.93±0.72 | ＜0.01 |

GLSZM, Gray-Level-Size-Zone-Matrix; GLRLM, Gray-Level-Run-Length-Matrix; NGTDM, Neighborhood gray-tone difference matrix; GLCM, Grey-Level-Co-occurrence-Matrix.
